# Effect of Bang® Pre-Workout Master Blaster® combined with four weeks of resistance training on lean body mass, maximal strength, mircoRNA expression, and serum IGF-1 in men: a randomized, double-blind, placebo-controlled trial

**DOI:** 10.1186/s12970-019-0310-y

**Published:** 2019-11-19

**Authors:** Neil A. Schwarz, Sarah K. McKinley-Barnard, Zachary J. Blahnik

**Affiliations:** Department of Health, Kinesiology, and Sport, University of South Alabama, Mobile, AL 36688 USA

**Keywords:** microRNA, Skeletal muscle, Resistance exercise, Hypertrophy, Betaine, Creatine, Caffeine, Branched-chain amino acids, Citrulline, Beta-alanine

## Abstract

**Background:**

The aim of the current study was to determine if 4 weeks of consumption of Bang® Pre-Workout Master Blaster® (BMB; Vital Pharmaceuticals Inc., Weston, FL) combined with resistance training resulted in greater increases in muscle mass and maximal strength compared with resistance training combined with placebo (PLA). Additionally, we aimed to determine if BMB ingestion combined with resistance training preferentially altered resting skeletal muscle expression of microRNAs (miRs) or resting serum insulin-like growth factor (IGF-1).

**Methods:**

Sixteen recreationally-active men completed the study. The study employed a block-randomized, double-blind, placebo-controlled, parallel design. Participants completed two testing sessions separated by 4 weeks of resistance exercise combined with daily supplementation of BMB or PLA. At each testing session, hemodynamics, body composition, and muscle and blood samples were obtained followed by strength assessments of the lower- and upper-body via measurement of squat and bench press one-repetition maximum (1-RM), respectively. A separate general linear model was utilized for analysis of each variable to determine the effect of each supplement (between-factor) over time (within-factor) using an a priori probability level of ≤0.05.

**Results:**

No significant effects were observed for dietary intake, hemodynamics, fat mass, body fat percentage, or serum IGF-1. A greater increase in total body mass (3.19 kg, 95% CI, 1.98 kg, 4.40 kg vs. 0.44 kg, 95% CI, − 0.50 kg, 1.39 kg) and lean body mass (3.15 kg, 95% CI, 1.80 kg, 4.49 kg vs. 0.89 kg, 95% CI, − 0.14 kg, 1.93 kg) was observed for the BMB group compared with PLA (*p* <  0.01). A significant increase over time was observed for miR-23a (*p* = 0.02) and miR-23b (*p* = 0.05) expression. A greater increase in squat 1-RM was observed for the BMB group (23.86 kg, 95% CI, 16.75 kg, 30.97 kg) compared with the PLA group (14.20 kg, 95% CI, 7.04 kg, 21.37 kg, *p* = 0.04).

**Conclusions:**

BMB supplementation combined with resistance exercise training for 4 weeks resulted in superior adaptations in maximal strength and LBM compared with resistance training with a placebo. No adverse resting hemodynamic or clinical blood safety markers were observed as a result of BMB supplementation. The superior outcomes associated with BMB supplementation could not be explained by resting serum IGF-1 or the skeletal muscle miRs measured, although resting miR-23a and miR-23b expression both increased as a result of resistance training.

## Background

Resistance training is well-known to increase muscle mass and maximal strength [1], yet the mechanisms regulating the adaptive responses to resistance training are complex and not completely elucidated [2]. Resistance exercise stimulates robust changes in the transctriptome and translational activity within skeletal muscle fibers which, with repeated stimulation, lead to changes in fiber size and function. In addition, resistance training can induce changes in basal epigenetic activity favorable for hypertrophy [3]. One epigenetic mechanism altered by resistance exercise is the expression of microRNA (miR), small non-coding RNA molecules with the ability to alter expression of target mRNA through degradation or translation inhibition [3, 4]. Powerlifters demonstrate differential basal expression of miRs in skeletal muscle compared with healthy controls [5]. D’Souza et al. [5] were able to discriminate between skeletal muscle from powerlifters and healthy controls with 100% accuracy using miR-126, −23b, − 16, −23a, and -15a as determinants suggesting a role of these miRs in the regulation of resistance training adaptations.

Because of the complexity of the regulatory processes involved in resistance training adaptations, the rate of these adaptations can potentially be influenced by many factors [2, 6]. One factor especially important in dictating adaptations is dietary intake [7]; thus, ingestion of multi-ingredient pre-workout supplements (MIPS) prior to resistance exercise has become commonplace due to the belief that they will provide support for an optimal adaptive response [8, 9]. However, the type and amount of ingredients in different commercially-available MIPS vary considerably making it important for the purported claims of each product to be validated. Additionally, the effect of MIPS ingestion on the basal expression of miRs associated with skeletal muscle adaptations to resistance training is relatively unexplored.

We previously reported acute ingestion of Bang® Pre-Workout Master Blaster® (BMB; Vital Pharmaceuticals Inc., Weston, FL), a commercially-available MIPS, to increase lower-body power and muscular endurance [10]. Additionally, serum insulin-like growth factor-1 (IGF-1) and human growth hormone (HGH) were preferentially increased after exercise with acute BMB ingestion compared with exercise and placebo [10]. The primary aim of the current study was to follow-up on our previous findings and determine if chronic consumption of BMB combined with resistance training resulted in greater increases in muscle mass and maximal strength compared with resistance training combined with placebo. Additionally, we aimed to determine if BMB ingestion combined with resistance training preferentially alters resting skeletal muscle expression of miR-126, miR-23b, miR-16, miR-23a, and miR-15a or resting serum IGF-1. We hypothesized that chronic ingestion of BMB combined with 4 weeks of resistance training would result in preferential changes in lean body mass, maximal strength, resting serum IGF-1, and resting miR expression compared with resistance training combined with ingestion of a placebo.

## Methods

### Experimental design

**Fig. 1:**
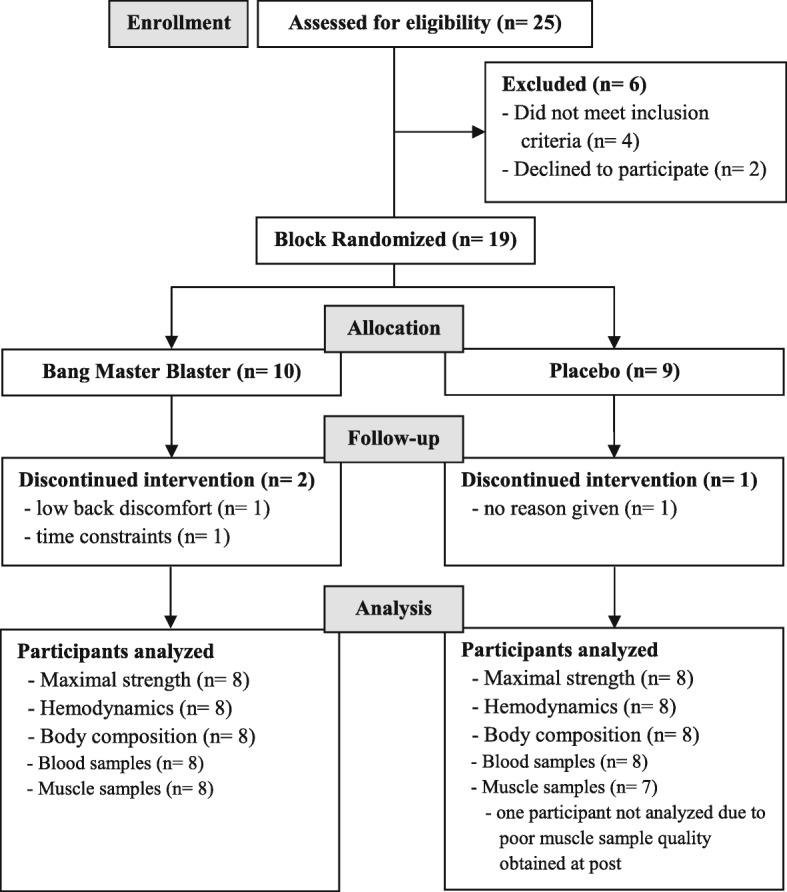
CONSORT Flow Diagram

The study employed a block-randomized, double-blind, placebo-controlled, parallel design (Fig. 1). Participants completed an entry session during which the requirements of the study were explained, informed consent was obtained, and testing exercises were familiarized. Participants completed two testing sessions (Pre and Post) in the morning separated by 4 weeks of resistance exercise combined with supplementation of BMB or placebo (PLA). Participants were instructed to complete a 3-day diet recall, fast for at least 10 h, and refrain from exercise for at least 48 h prior to each testing session. On the morning of the testing session, participants reported to the human performance laboratory where height and body mass measurements were obtained. Participants then rested for 5 min while seated in a chair after which hemodynamics were measured. After hemodynamic measurements, the participants completed a body composition assessment using dual-energy x-ray absorptiometry (DXA). After the DXA scan, a venous blood sample and skeletal muscle biopsy sample were obtained. Lastly, the participants completed a maximal strength assessment of the lower- and upper-body via measurement of squat and bench press one-repetition maximum (1-RM), respectively. Participants were block-randomized to BMB or PLA based on resistance training status and maximal squat strength. Post-testing sessions were identical to the pre-testing sessions and were performed at approximately the same time of day as the pre-testing session for each participant.

### Participant characteristics

Sixteen recreationally-active men completed the study (BMB group: *n* = 8, age = 22.5 ± 2.9 years; height = 181.7 ± 9.2 cm; PLA group: n = 8, age = 22.5 ± 3.1 years; height = 175.3 ± 8.1 cm). Each group had a large, but similar, variance of resistance training experience. The average self-reported resistance training experience was 3.19 ± 2.96 years with a range of less than 1 year of experience (*n* = 3) to 8 years of experience for the PLA group and 2.94 ± 2.44 years with a range of less than 1 year of experience (also n = 3) to 7 years of experience. Participants did not consume dietary supplements (except multivitamins/multiminerals, caffeine, and/or protein powder) for at least 1 month prior to entering the study. Participants completed a health history questionnaire and a physical activity questionnaire prior to completing the study to assess health status and exercise training experience. Exclusion criteria included a history of or current health condition including diabetes, cardiovascular disease, arrhythmias, thyroid disease, hypogonadism, pulmonary disease, liver or kidney disease, musculoskeletal disorders, neuromuscular or neurological diseases, autoimmune disease, cancer, peptic ulcers, or anemia. Participants were familiarized to the study protocol via a verbal and written explanation outlining the study design and signed an informed consent document approved by the University of South Alabama Institutional Review Board (IRBNet #: 966357; Approval Date: 10/11/2016). All experimental procedures involved in the study conformed to the ethical consideration of the Declaration of Helsinki.

#### Supplementation protocol

**Fig. 2:**
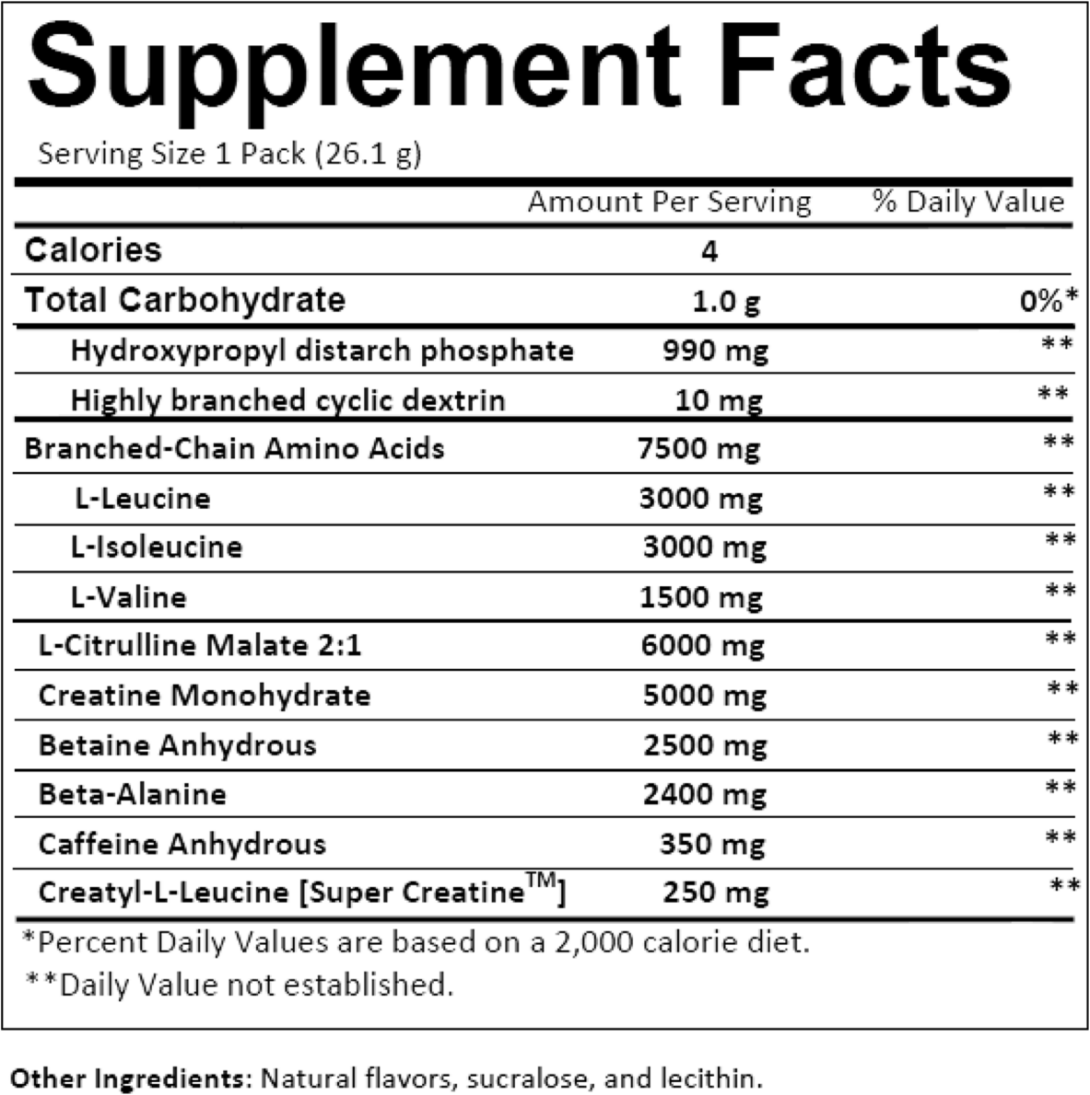
Supplement Facts for Bang® Pre-Workout Master Blaster®. Contrary to the label, the estimated energy value for the supplement is 34 kcal

Participants began consuming their assigned supplement on the day following the pre-testing session and consumed the last serving on the morning of the day prior to the post-testing session. Participants consumed one serving (26.1 g) of BMB (Fig. 2; energy value of 34 kcal) or PLA 30 min prior to the onset of each resistance exercise training session. On non-workout days, participants consumed their assigned supplement in the morning. The placebo being used for this study was formulated by Vital Pharmaceuticals Inc. (Weston, FL) and contained Fibersol®-2 with a similar look and flavor profile to that of the supplement. The BMB and PLA supplements were provided to participants as a pre-measured powder that was mixed with water prior to ingestion. The placebo and supplement were provided by Vital Pharmaceuticals Inc. in identical packages marked as “A” or “B”, and the contents of each were unknown until after data collection. Reported compliance for supplement ingestion was 100%.

### Resistance training protocol

The resistance training program was initiated 2 to 3 days after the pre-testing session. Participants completed a four-week periodized resistance training program consisting of two lower-body and two upper-body sessions per week for a total of 16 sessions. Each resistance exercise session was supervised by study personnel and consisted of seven exercises with 60 to 120 s rest between sets. The resistance training protocol is outlined in Table 1.

**Table 1:** Outline of Resistance Training Protocol

Lower Body - Days 1 & 3 of each Week									
	Week 1		Week 2		Week 3		Week 4		
Exercise	Sets	Reps	Sets	Reps	Sets	Reps	Sets	Reps	Rest
Box Squat	2	8–12	3	8–12	3	6–8	4	3–5	120 s
Leg Press	2	15–20	3	15–20	3	12–15	4	12–15	120 s
Leg Extension^a^	2	8–10	3	8–10	3	6–8	4	6–8	60s
Leg Curl^b^	2	8–10	3	8–10	3	6–8	4	6–8	60s
Calf Raise^c^	2	12–15	3	12–15	3	10–12	4	10–12	60s
Decline Sit-ups^d^	2	AMRAP	3	AMRAP	3	AMRAP	4	AMRAP	60s
Walking Lunges	1	25 yds	2	25 yds	3	25 yds	3	25 yds	60s
Upper Body - Days 2 & 4 of each Week									
	Week 1		Week 2		Week 3		Week 4		
Exercise	Sets	Reps	Sets	Reps	Sets	Reps	Sets	Reps	Rest
Bench Press	2	8–12	3	8–12	3	6–8	4	3–5	120 s
Lat Pulldown	2	12–15	3	12–15	3	8–10	4	8–10	120 s
Shoulder Press^a^	2	10–12	3	10–12	3	8–10	4	8–10	60s
Cable Row^b^	2	10–12	3	10–12	3	8–10	4	8–10	60s
Triceps Pushdown^c^	2	12–15	3	12–15	3	10–12	4	10–12	60s
Biceps Curl^d^	2	12–15	3	12–15	3	10–12	4	10–12	60s
Shrugs	1	20–25	2	20–25	3	15–20	3	15–20	60s

AMRAP = as many reps as possible; exercises denoted with an ^a^ and ^b^ were performed in an alternated fashion; exercises denoted with an ^c^ and ^d^ were performed in an alternated fashion

### Hemodynamic assessment

Heart rate and blood pressure were determined in the seated position after resting for 10 min. Heart rate was measured by palpation of the radial artery for 30 s. Blood pressure was assessed with a mercurial sphygmomanometer and stethoscope (Welch Allyn, Skaneateles Falls, NY) using standard procedures.

### Anthropometric and body composition assessment

Total body mass (kg) and height (cm) were determined using a calibrated scale and stadiometer (Seca model 700, Seca Corporation, Chino, CA). Body composition was measured by DXA (Horizon Wi, Hologic, Bedford, MA, USA).

### Maximal strength assessment

Assessment of maximal strength was determined using a 1-RM test for the squat exercise followed by the bench press exercise at both the pre- and post-testing sessions. The procedures for obtaining the 1-RM measurement were the same for both exercises. Participants warmed-up by cycling on an Airdyne bicycle (Schwinn, Vancouver, WA) for 5 min at a self-determined pace followed by completion of 8 to 10 repetitions at approximately 50% of estimated 1-RM. The participant rested for approximately 2 minutes and then completed 3–5 repetitions at approximately 70% of estimated 1-RM. The weight was then increased conservatively and the participant attempted to lift the weight for one repetition. If the lift was successful, the participant rested for 2 minutes before testing the next weight increment. This procedure continued until the participant failed to complete the lift successfully. The 1-RM was recorded as the maximum weight that the participant was able to lift for one repetition.

The squat exercise was performed using a Smith machine (Maxicam, Muscle Dynamics, Paramount, CA) to help standardize form. In addition, squats were performed down to a squat box (Elitefts™, London, OH) to standardize squat depth to 90 degrees of knee flexion for all participants. For the squat to be considered successful, participants were required to squat down until lightly touching the box before beginning the concentric portion of the lift. The bench press exercise was performed in a power rack using an adjustable bench (Hammer Strength, Life Fitness, Rosemont, IL). Participants were required to touch the chest with the barbell before performing the concentric portion of the lift in order to be considered successful.

### Venous blood sampling and skeletal muscle biopsies

Venous blood from the antecubital vein was collected at rest using a Vacutainer apparatus and needle (Becton, Dickinson and Company, Franklin lakes, NJ). Blood samples used for complete blood count (CBC) analysis were collected in EDTA tubes and inverted to prevent clotting. Blood samples used for comprehensive metabolic panel (CMP) and IGF-1analysis were collected using serum separator tubes, allowed to stand at room temperature for 10 min, and then centrifuged. CBC and CMP analyses were outsourced to LabCorp Inc., Birmingham, AL. Serum used for the IGF-1 assay was removed and aliquoted into 1.5 mL tubes and immediately frozen at − 80 °C for later analysis.

Percutaneous muscle biopsies (~ 30 mg) were obtained at rest from the middle portion of the vastus lateralis muscle at the midpoint between the patella and the greater trochanter of the femur at a depth between 1 and 2 cm based on previously-used procedures [11]. The same leg and general location (determined by pre-biopsy markings) was biopsied at each testing session. The biopsy area was shaved clean of leg hair and cleaned with rubbing alcohol. A small area of the cleaned skin ~ 2 cm in diameter was anesthetized with a 1.5 mL subcutaneous injection of 1% lidocaine hydrochloride (Hospira, Lake Forest, IL). After, the biopsy site was further cleansed by swabbing the area with povidine-iodine. Once anesthetized, a pilot hole was created using a sterile 12-gauge needle followed by insertion of a 14-gauge fine needle aspiration biopsy instrument (Pro-Mag Ultra Automatic Biopsy Instrument, Argon Medical, Gainesville, FL) was inserted into the skin at an approximate depth of 1 cm to extract the muscle sample using three passes. After removal, adipose tissue was trimmed from the muscle specimens. Specimens were immediately immersed in 500 μL of RNAlater stabilization solution (Life Technologies, Carlsbad, CA) and stored at − 80 °C for later analysis.

### Serum IGF-1 analysis

Serum samples were analyzed in duplicate for IGF-1 (ALPCO, Salem, NH) using enzyme-linked immunosorbent assay (ELISA) following the manufacturer’s supplied protocol and absorbances were measured at a wavelength of 450 nm using a microplate reader (SpectraMax Plus 384, Molecular Devices, Sunnyvale, CA). Concentrations of the unknown samples were calculated using data reduction software (SoftMax Pro, Molecular Devices, Sunnyvale, CA). Serum IGF-1 assays were performed using a 1:21 sample dilution with an intra-assay coefficient of variance of 7.6%.

### Skeletal muscle microRNA analyses

Total RNA was isolated from muscle samples using the mirVana PARIS kit according to manufacturer’s specifications (Life Technologies, Carlsbad, CA) as previously described [12]. cDNA synthesis and real-time polymerase chain reaction (RT-PCR) were performed using the qScript® microRNA cDNA Synthesis Kit (QuantaBio, Beverly, MA) and PerfeCTa® SYBR® Green SuperMix (QuantaBio, Beverly, MA). Primers for miRs (miR-15a-5p, miR-23a-5p, miR-23b-5p, miR-126-3p, miR-16-5p, miR-361-5p, miR-320a, miR-186-5p; Additional file 1: Table S1) were commercially synthesized (Integrated DNA Technologies, Coralville, IA). Reactions totaling 25 μL consisting of 5 μL of miRNA cDNA template, 12.5 μL of PerfeCta SYBR Green SuperMix (Quantabio, Beverly, MA), 0.5 μL of the PerfeCTa Universal PCR Primer, 0.5 μL of the target miRNA primer, and 6.5 μL of nuclease-free water were added to each well. Each reaction was amplified using RT-PCR on a qTower 2.2 (Analytik Jena US LLC, Beverly, MA). The amplification profile was run for an initial pre-incubation/activation phase at 95 °C for 2 min and then for 40 cycles of 95 °C for 5 s and 60 °C for 30 s according to manufacturer specifications (QuantaBio, Beverly, MA). Fluorescence was measured after each cycle. Relative miR expression was determined by the 2^-ΔΔCt^ method using the geometric mean of three miRNAs (miR-361-5p, miR-320a, miR-186-5p) as a reference [5, 13, 14]. Data were expressed with post-testing levels normalized to pre-testing levels for each group. Intra-assay coefficients of variance for miR-186, − 320, − 361, − 15, − 16, −23a, −23b, and − 126 were 0.51, 0.82, 0.94, 0.79, 0.67, 0.95, 0.56, and 0.86%, respectively.

### Dietary analyses

Dietary intake data for (24-h recalls) were collected and analyzed using the Automated Self-Administered 24-h (ASA24) Dietary Assessment Tool, version 2016, developed by the National Cancer Institute, Bethesda, MD [15]. The participants’ diets were not standardized, but participants were instructed not to change their dietary habits during the course of the study. A 3-day diet recall was completed by the participants before each testing session.

### Statistical analyses

Data for each group at each time point were checked for normality of distribution using the Shapiro-Wilk test. Of the 46 variables analyzed statistically, 11 had at least one dataset of each group at either time point not normally distributed according to the Shapiro-Wilk test (mean cell hemoglobin, monocyte count, eosinophil count, basophil count, glucose, potassium, bilirubin, aspartate aminotransferase, alanine aminotransferase, miR-15, and miR-23a). Data for these variables were first analyzed non-parametrically and resulted in similar outcomes to the parametric tests employed; thus, results of the parametric tests are presented. A separate general linear model was utilized for analysis of each variable to determine the effect of each supplement (between-factor) over time (within-factor) on hemodynamics, body composition, maximal strength, serum IGF-1, skeletal muscle miRNA expression, blood safety markers, and dietary intake. Effect sizes for interaction effects were calculated as partial eta-squared (*ƞ*^2^). If no significant interaction was observed, main effects were analyzed using paired samples *t* test for time comparisons and independent samples *t* test for group comparisons. If a significant interaction was observed, simple main effects were analyzed using paired samples *t* test for time comparisons for each group and independent samples *t* test for group comparisons at each time point. Effect sizes for main effects and simple main effects were calculated as Cohen’s *d* using Excel (Microsoft Corp., Redmond, WA). Statistical analyses were performed using SPSS Statistics 22.0 (IBM Corp.; Armonk, NY) and an a priori probability level of ≤0.05 was adopted.

## Results

### Dietary analyses

No significant interaction effects were observed for kilocalorie (*p* = 0.98; partial *n*^2^ <  0.01), protein (*p* = 0.57; partial *n*^2^ = 0.02), fat (*p* = 0.60; partial *n*^2^ = 0.02), or carbohydrate (*p* = 0.47; partial *n*^2^ = 0.04) intake (Table 2). No significant differences for the main effect of time were observed for kilocalorie (*p* = 0.87; Cohen’s *d* = 0.05), protein (*p* = 0.82; Cohen’s *d* = 0.07), fat (*p* = 0.38; Cohen’s *d* = 0.25), or carbohydrate (*p* = 0.58; Cohen’s *d* = 0.16) intake. No significant differences for the main effect of group were observed for kilocalorie (*p* = 0.61; Cohen’s *d* = 0.18), protein (*p* = 0.29; Cohen’s *d* = 0.37), fat (*p* = 0.96; Cohen’s *d* = 0.03), or carbohydrate (*p* = 0.99; Cohen’s *d* < 0.01) intake.

**Table 2:** Reported Average Total Kilocalorie and Macronutrient Intake for Each Group and Time Point

Variable	Time Point	PLA	BMB
Kilocalories (Kcal/day)	PRE	2281 ± 598	2406 ± 773
	POST	2310 ± 826	2443 ± 724
Protein (g/day)	PRE	113 ± 41	123 ± 61
	POST	103 ± 33	127 ± 41
Fat (g/day)	PRE	82 ± 20	88 ± 28
	POST	96 ± 49	91 ± 42
Carbohydrate (g/day)	PRE	268 ± 96	249 ± 50
	POST	263 ± 101	282 ± 100

Data presented as mean ± standard deviation

### Hemodynamics

**Fig. 3:**
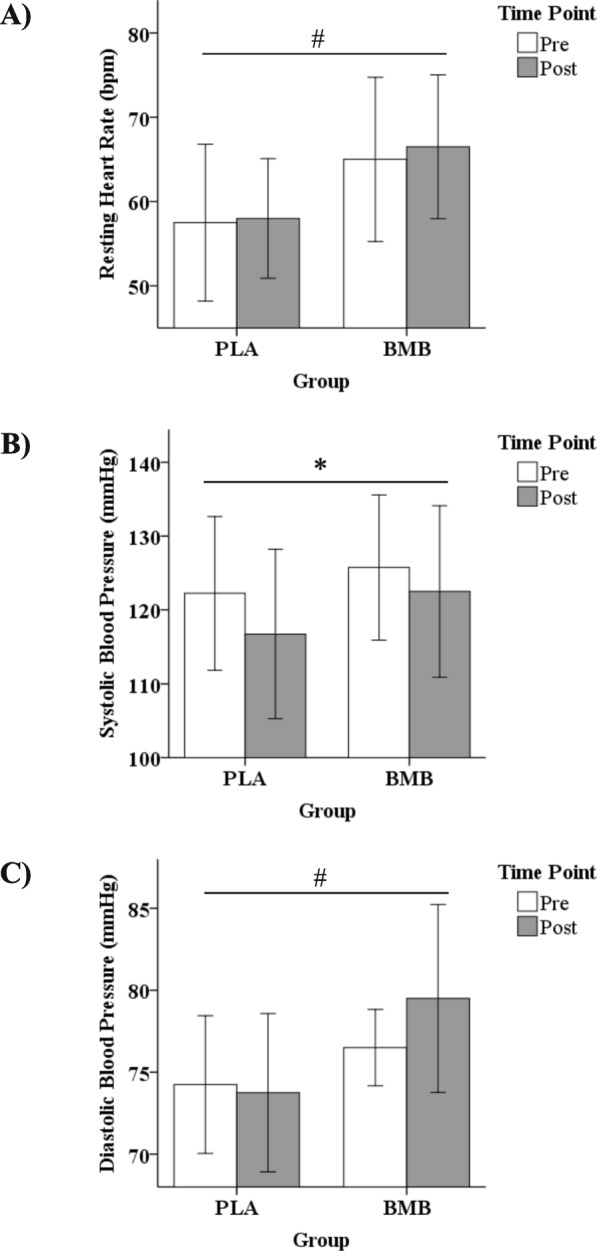
Mean ± standard deviation for **a**) resting heart rate, **b**) systolic blood pressure, and **c**) diastolic blood pressure at the Pre and Post time points for each group. Note. Whisker bars represent the standard deviation; PLA = placebo; BMB = Bang Master Blaster. * denotes statistically significant for the main effect of time. # denotes statistically significant for the main effect of group

No significant interaction effects were observed for heart rate (*p* = 0.77; partial *n*^2^ = 0.03), systolic blood pressure (*p* = 0.59; partial *n*^2^ = 0.02), or diastolic blood pressure (*p* = 0.17; partial *n*^2^ = 0.13; Fig. 3a-c). No significant differences for the main effect of time were observed for heart rate (*p* = 0.54; Cohen’s *d* = 0.11) or diastolic blood pressure (*p* = 0.34; Cohen’s *d* = 0.25). A significant decrease in systolic blood pressure was observed for the main effect of time (*p* = 0.05; Cohen’s *d* = 0.37). No significant differences for the main effect of group were observed for systolic blood pressure (*p* = 0.23; Cohen’s *d* = 0.43). A significant difference for the main effect of group was observed for heart rate (*p* = 0.01; Cohen’s *d* = 0.95) and diastolic blood pressure (*p* = 0.02; Cohen’s *d* = 0.90) with both significantly higher for the BMB group.

### Body composition

**Fig. 4:**
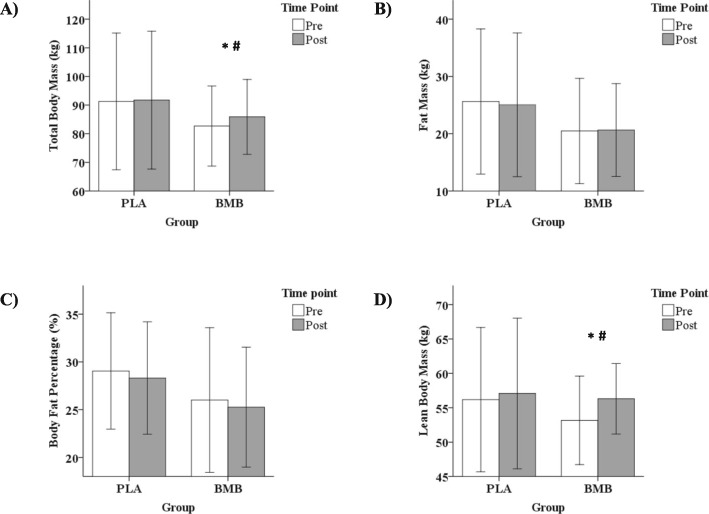
Mean ± standard deviation for **a**) total body mass, **b**) fat mass, **c**) body fat percentage, and **d**) lean body mass at the Pre and Post time points for each group. Note. Whisker bars represent the standard deviation; PLA = placebo; BMB = Bang Master Blaster; * denotes statistically significant increase from Pre to Post; # denotes statistically greater increase from Pre to Post for BMB compared with PLA

A significant interaction between group and time was observed for total body mass (TBM; *p* < 0.01; partial *n*^2^ = 0.56). A significant increase in TBM was observed over time for the BMB group (+ 3.19 kg, 95% CI, 1.98 kg, 4.40 kg, *p* < 0.001; Cohen’s *d* = 0.24), but not the PLA group (+ 0.44 kg, 95% CI, − 0.50 kg, 1.39 kg, *p* = 0.30; Cohen’s *d* = 0.02). No difference between groups was observed for TBM at the pre-testing (*p* = 0.39; Cohen’s *d* = 0.44) or post-testing (*p* = 0.56; Cohen’s *d* = 0.30) time points (Fig. 4a).

No significant interaction effect was observed for fat mass (*p* = 0.39; partial *n*^2^ = 0.05) or body fat % (*p* = 0.99; partial *n*^2^ < 0.01). The main effect of time was not significant for fat mass (*p* = 0.64; Cohen’s *d* = 0.02) or body fat % (*p* = 0.11 Cohen’s *d* = 0.11). Likewise, the main of effect of group was not significant for fat mass (*p* = 0.39; Cohen’s *d* = 0.46) or body fat % (*p* = 0.36; Cohen’s *d* = 0.49; Fig. 4b and c).

A significant interaction between group and time was observed for LBM (*p* < 0.01; partial *n*^2^ = 0.41). A significant increase in LBM was observed over time for the BMB group (+ 3.15 kg, 95% CI, 1.80 kg, 4.49 kg, *p* < 0.01; Cohen’s *d* = 0.54), but not PLA (+ 0.89 kg, 95% CI, − 0.14 kg, 1.93 kg, *p* = 0.08; Cohen’s *d* = 0.08). No difference between groups was observed for LBM at the pre-testing (*p* = 0.50; Cohen’s *d* = 0.35) or post-testing (*p* = 0.86; Cohen’s *d* = 0.09) time points (Fig. 4d).

### Maximal strength

**Fig. 5:**
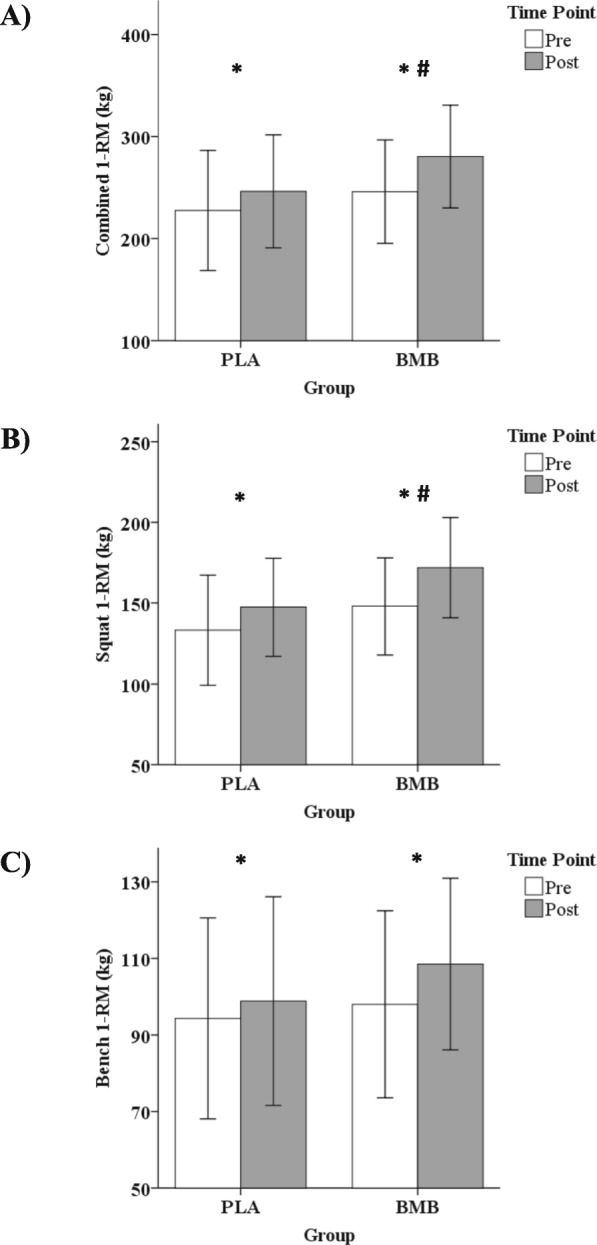
Mean ± standard deviation for **a**) combined 1-RM, **b**) squat 1-RM, and **c**) bench press 1-RM at the Pre and Post time points for each group. Note. Whisker bars represent the standard deviation; PLA = placebo; BMB = Bang Master Blaster; * denotes statistically significant increase from Pre to Post; # denotes statistically greater increase from Pre to Post for BMB compared with PLA

A significant interaction between group and time was observed (*p* = 0.02; partial *n*^2^ = 0.32) for combined strength (squat + bench 1-RM). A significant increase in combined strength was observed over time for the BMB group (+ 34.38 kg, 95% CI, 21.75 kg, 47.00 kg, *p* < 0.01; Cohen’s *d* = 0.68) and the PLA group (+ 18.75 kg, 95% CI, 11.88 kg, 25.62 kg, *p* < 0.01; Cohen’s *d* = 0.33). No difference between groups was observed for combined strength at the pre-testing (*p* = 0.51; Cohen’s *d* = 0.34) or post-testing (*p* = 0.22; Cohen’s *d* = 0.64) time points (Fig. 5a).

Individually, a significant interaction between group and time was observed for squat 1-RM (*p* = 0.04; partial *n*^2^ = 0.27). A significant increase in squat 1-RM was observed over time for the BMB group (+ 23.86 kg, 95% CI, 16.75 kg, 30.97 kg, *p* < 0.01; Cohen’s *d* = 0.78) and the PLA group (+ 14.20 kg, 95% CI, 7.04 kg, 21.37 kg, *p* < 0.01; Cohen’s *d* = 0.44). No difference between groups was observed for squat 1-RM at the pre-testing (*p* = 0.37; Cohen’s *d* = 0.46) or post-testing (*p* = 0.13; Cohen’s *d* = 0.80) time points (Fig. 5b). No significant interaction between group and time was observed for bench press 1-RM (*p* = 0.08; partial *n*^2^ = 0.20). A significant increase was observed for the main effect of time (*p* < 0.01; Cohen’s *d* = 0.31), with no significant difference observed for the main effect of group (*p* = 0.45; Cohen’s *d* = 0.27; Fig. 5c).

### Whole blood and serum clinical chemistry markers

A significant interaction between group and time was observed for white blood cell count (*p* = 0.04; partial *n*^2^ = 0.28), platelet count (*p* < 0.01; partial *n*^2^ = 0.42), lymphocyte count (*p* < 0.01; partial *n*^2^ = 0.47), creatinine (*p* < 0.01; partial *n*^2^ = 0.48), and calcium (*p* = 0.03; partial *n*^2^ = 0.31). White blood cell count (*p* = 0.04; Cohen’s *d* = 0.63), platelet count (*p* = 0.05; Cohen’s *d* = 0.25), and lymphocyte count (*p* = 0.01; Cohen’s *d* = 0.40) decreased in the PLA group over time. No significant effect of time was observed for PLA for creatinine (*p* = 0.96; Cohen’s *d* = 0.01) or calcium (*p* = 0.23; Cohen’s *d* = 0.64). Lymphocyte count (*p* = 0.05; Cohen’s *d* = 0.70) and creatinine (*p* < 0.01; Cohen’s *d* = 0.96) increased over time in the BMB group. No significance for time was observed in the BMB group for white blood cell count (*p* = 0.27; Cohen’s *d* = 0.60), platelet count (*p* = 0.06; Cohen’s *d* = 0.32), or calcium (*p* = 0.07; Cohen’s *d* = 0.54). At the pre-testing time point, lymphocyte count (*p* = 0.05; Cohen’s *d* = 1.07) was significantly higher for the PLA group, with no significant difference between groups for white blood cell count (*p* = 0.38; Cohen’s *d* = 0.44), platelet count (*p* = 0.74; Cohen’s *d* = 0.17), creatinine (*p* = 0.07; Cohen’s *d* = 0.98), or calcium (*p* = 0.82; Cohen’s *d* = 0.09). At the post-testing time point, serum creatinine was significantly higher in the BMB group (*p* < 0.01; Cohen’s *d* = 1.64); whereas, calcium was significantly higher in the PLA group (*p* = 0.02; Cohen’s *d* = 1.35). No significant difference between groups was observed for white blood cell count (*p* = 0.13; Cohen’s *d* = 0.81), platelet count (*p* = 0.16; Cohen’s *d* = 0.74), or lymphocyte count (*p* = 0.83; Cohen’s *d* = 0.11) at the post-testing time point,

No significant interaction between group and time was observed for red blood cell count (*p* = 0.18; partial *n*^2^ = 0.12), hemoglobin (*p* = 0.41; partial *n*^2^ = 0.05), hematocrit (*p* = 0.65; partial *n*^2^ = 0.02), mean corpuscular volume (*p* = 0.36; partial *n*^2^ = 0.06), mean cell hemoglobin (*p* = 0.19; partial *n*^2^ = 0.12), mean corpuscular hemoglobin concentration (*p* = 0.84; partial *n*^2^ < 0.01), neutrophil count (*p* = 0.48; partial *n*^2^ = 0.04), monocyte count (*p* = 0.14; partial *n*^2^ = 0.15), eosinophil count (*p* = 0.12; partial *n*^2^ = 0.16), basophil count (*p* = 0.33; partial *n*^2^ = 0.07), glucose (*p* = 0.40; partial *n*^2^ = 0.05), blood urea nitrogen (*p* = 0.15; partial *n*^2^ = 0.14), sodium (*p* = 0.46; partial *n*^2^ = 0.04), potassium (*p* = 0.24; partial *n*^2^ = 0.10), chloride (*p* = 0.42; partial *n*^2^ = 0.05), carbon dioxide (*p* = 0.75; partial *n*^2^ = 0.01), protein (*p* = 0.80; partial *n*^2^ = 0.01), albumin (*p* = 0.83; partial *n*^2^ < 0.01), globulin (*p* = 0.61; partial *n*^2^ = 0.02), albumin/globulin ratio (*p* = 0.56; partial *n*^2^ = 0.03), bilirubin (*p* = 0.28; partial *n*^2^ = 0.08), alkaline phosphatase (*p* = 0.25; partial *n*^2^ = 0.09), aspartate aminotransferase (*p* = 0.41; partial *n*^2^ = 0.05), or alanine aminotransferase (*p* = 0.46; partial *n*^2^ = 0.04). No significance was observed for the main effect of time for red blood cell count (*p* = 0.63; Cohen’s *d* = 0.06), hemoglobin (*p* = 0.99; Cohen’s *d* < 0.01), hematocrit (*p* = 0.37; Cohen’s *d* = 0.15), mean corpuscular volume (*p* = 0.11; Cohen’s *d* = 0.26), mean cell hemoglobin (*p* = 0.85; Cohen’s *d* = 0.02), mean corpuscular hemoglobin concentration (*p* = 0.27; Cohen’s *d* = 0.30), neutrophil count (*p* = 0.38; Cohen’s *d* = 0.23), monocyte count (*p* = 0.38; Cohen’s *d* = 0.22), eosinophil count (*p* = 0.06; Cohen’s *d* = 0.44), basophil count (*p* = 0.33; Cohen’s *d* = 0.19), blood urea nitrogen (*p* = 0.73; Cohen’s *d* = 0.07), sodium (*p* = 0.09; Cohen’s *d* = 0.51), potassium (*p* = 0.29; Cohen’s *d* = 0.40), chloride (*p* = 0.41; Cohen’s *d* = 0.26), carbon dioxide (*p* = 0.11; Cohen’s *d* = 0.67), globulin (*p* = 0.13; Cohen’s *d* = 0.52), albumin/globulin ratio (*p* = 0.33; Cohen’s *d* = 0.23), bilirubin (*p* = 0.95; Cohen’s *d* = 0.02), alkaline phosphatase (*p* = 0.49; Cohen’s *d* = 0.05), aspartate aminotransferase (*p* = 0.44; Cohen’s *d* = 0.25), or alanine aminotransferase (*p* = 0.48; Cohen’s *d* = 0.20). Likewise, no significance was observed for the main effect of group for red blood cell count (*p* = 0.09; Cohen’s *d* = 0.63), hemoglobin (*p* = 0.18; Cohen’s *d* = 0.49), hematocrit (*p* = 0.13; Cohen’s *d* = 0.55), mean corpuscular volume (*p* = 0.75; Cohen’s *d* = 0.11), mean cell hemoglobin (*p* = 0.46; Cohen’s *d* = 0.27), mean corpuscular hemoglobin concentration (*p* = 0.67; Cohen’s *d* = 0.15), neutrophil count (*p* = 0.16; Cohen’s *d* = 0.51), monocyte count (*p* = 0.32; Cohen’s *d* = 0.36), eosinophil count (*p* = 0.07; Cohen’s *d* = 0.68), basophil count (*p* = 0.16; Cohen’s *d* = 0.51), glucose (*p* = 0.47; Cohen’s *d* = 0.26), blood urea nitrogen (*p* = 0.09; Cohen’s *d* = 0.63), sodium (*p* = 0.12; Cohen’s *d* = 0.57), potassium (*p* = 0.54; Cohen’s *d* = 0.22), chloride (*p* = 0.57; Cohen’s *d* = 0.20), carbon dioxide (*p* = 0.43; Cohen’s *d* = 0.28), protein (*p* = 0.85; Cohen’s *d* = 0.07), albumin (*p* = 0.61; Cohen’s *d* = 0.18), globulin (*p* = 0.64; Cohen’s *d* = 0.17), albumin/globulin ratio (*p* = 0.60; Cohen’s *d* = 0.19), alkaline phosphatase (*p* = 0.31; Cohen’s *d* = 0.36), aspartate aminotransferase (*p* = 0.49; Cohen’s *d* = 0.25), or alanine aminotransferase (*p* = 0.51; Cohen’s *d* = 0.24). A significant main effect for time was observed for glucose (*p* = 0.01; Cohen’s *d* = 0.72) protein (*p* = 0.02; Cohen’s *d* = 0.71), and albumin (*p* = 0.03; Cohen’s *d* = 0.41). Glucose and albumin were significantly increased at the post-testing time point compared with pre-testing; whereas, protein decreased from pre-to-post-testing. A significant main effect for group was observed for bilirubin (*p* = 0.04; Cohen’s *d* = 0.79) with the PLA group significantly higher compared with the BMB group. Although some statistical changes were observed, all mean values were still within the normal clinical reference range (Table 3).

**Table 3:** Complete Blood Count (CBC) and Comprehensive Metabolic Panel (CMP) Results for Each Variable and Time Point

Variable	Normal Range	Time Point	PLA	BMB
WBC (10^3^/μL)	3.4–10.8	PRE	5.41 ± 0.76	5.08 ± 0.73
		POST	4.94 ± 0.74*	5.46 ± 0.52
RBC (10^6^/μL)	4.14–5.80	PRE	5.13 ± 0.38	4.97 ± 0.31
		POST	5.17 ± 0.37	4.90 ± 0.37
Hemoglobin (g/dL)	12.6–17.7	PRE	14.73 ± 0.96	14.38 ± 1.03
		POST	14.83 ± 0.78	14.28 ± 1.02
Hematocrit (%)	37.5–51.0	PRE	44.78 ± 2.52	43.43 ± 3.19
		POST	45.45 ± 0.08	43.64 ± 2.95
MCV (fL)	79–97	PRE	87.62 ± 3.11	87.50 ± 5.95
		POST	88.13 ± 4.76	89.25 ± 3.73
MCH (pg)	26.6–33.0	PRE	28.75 ± 1.47	28.96 ± 1.99
		POST	28.54 ± 1.73	29.25 ± 2.02
MCHC (g/dL)	31.5–35.7	PRE	32.89 ± 0.93	33.10 ± 0.78
		POST	32.64 ± 1.04	32.74 ± 1.40
Platelets (10^3^/μL)	150–379	PRE	235.00 ± 45.16	241.88 ± 33.86
		POST	224.75 ± 37.31*	254.37 ± 42.99
Neutrophils (10^3^/μL)	1.4–7.0	PRE	2.69 ± 0.76	2.86 ± 0.48
		POST	2.44 ± 0.47	2.84 ± 0.57
Lymphocytes (10^3^/μL)	0.7–3.1	PRE	2.11 ± 0.45	1.63 ± 0.45
		POST	1.94 ± 0.39**	1.90 ± 0.31*
Monocytes (10^3^/μL)	0.1–0.9	PRE	0.48 ± 0.07	0.41 ± 0.08
		POST	0.43 ± 0.13	0.43 ± 0.05
Eosinophils (10^3^/μL)	0.0–0.4	PRE	0.13 ± 0.05	0.16 ± 0.07
		POST	0.14 ± 0.07	0.25 ± 0.19
Basophils (10^3^/μL)	0.0–0.2	PRE	0.00 ± 0.00	0.03 ± 0.05
		POST	0.01 ± 0.04	0.03 ± 0.05
Glucose (mg/dL)	65–99	PRE	89.38 ± 6.57	85.63 ± 7.95
		POST	93.13 ± 9.67	92.75 ± 6.02
BUN (mg/dL)	6–20	PRE	14.25 ± 4.71	15.75 ± 4.17
		POST	13.25 ± 4.06	17.38 ± 5.29
Creatinine (mg/dL)	0.76–1.27	PRE	1.00 ± 0.08	1.10 ± 0.12
		POST	1.00 ± 0.13	1.23 ± 0.15**^##^
Sodium (mmol/L)	136–144	PRE	143.75 ± 3.45	141.75 ± 2.92
		POST	141.88 ± 1.25	141.00 ± 1.77
Potassium (mmol/L):	3.5–5.2	PRE	4.43 ± 0.31	4.49 ± 0.27
		POST	4.44 ± 0.19	4.25 ± 0.33
Chloride (mmol/L):	96–106	PRE	101.13 ± 2.10	100.13 ± 2.10
		POST	101.13 ± 1.81	101.25 ± 2.66
CO_2_ (mmol/L)	18–29	PRE	22.13 ± 2.42	21.75 ± 2.05
		POST	24.00 ± 2.62	23.00 ± 2.39
Calcium (mg/dL)	8.7–10.2	PRE	9.46 ± 0.18	9.43 ± 0.41
		POST	9.56 ± 0.13^#^	9.23 ± 0.32
Protein (g/dL)	6.0–8.5	PRE	7.20 ± 0.48	7.15 ± 0.39
		POST	6.93 ± 0.14	6.93 ± 0.35
Albumin (g/dL)	3.5–5.5	PRE	4.74 ± 0.30	4.69 ± 0.39
		POST	4.61 ± 0.23	4.54 ± 0.43
Globulin (g/dL)	1.5–4.5	PRE	2.46 ± 0.26	2.46 ± 0.14
		POST	2.31 ± 0.22	2.39 ± 0.26
A/G Ratio	1.1–2.5	PRE	1.93 ± 0.21	1.91 ± 0.22
		POST	2.03 ± 0.30	1.94 ± 0.34
Bilirubin (mg/dL)	0.0–1.2	PRE	0.76 ± 0.52	0.58 ± 0.21
		POST	0.86 ± 0.49	0.46 ± 0.17
ALP (IU/L)	39–117	PRE	85.88 ± 17.38	77.38 ± 20.26
		POST	85.25 ± 20.39	79.88 ± 20.86
AST (IU/L)	0.0–40	PRE	26.63 ± 7.19	32.38 ± 19.43
		POST	26.87 ± 5.36	26.63 ± 7.84
ALT (IU/L)	0.0–44	PRE	23.13 ± 9.91	32.50 ± 39.99
		POST	23.38 ± 8.05	23.88 ± 9.22

Data presented as mean ± standard deviation. ^a^Denotes significant increase from PRE to POST for main effect of time. ^b^Denotes significant decrease from PRE to POST for main effect of time. ^†^Denotes significant group x time interaction. *Denotes significant difference (*p* ≤ 0.05) from PRE to POST for group. **Denotes significant difference (*p* < 0.01) from PRE to POST for group. ^#^Denotes significant difference (*p* ≤ 0.05) between groups at time point. ^##^Denotes significant difference (*p* ≤ 0.01) between groups at time point.

### Serum IGF-1

**Fig. 6:**
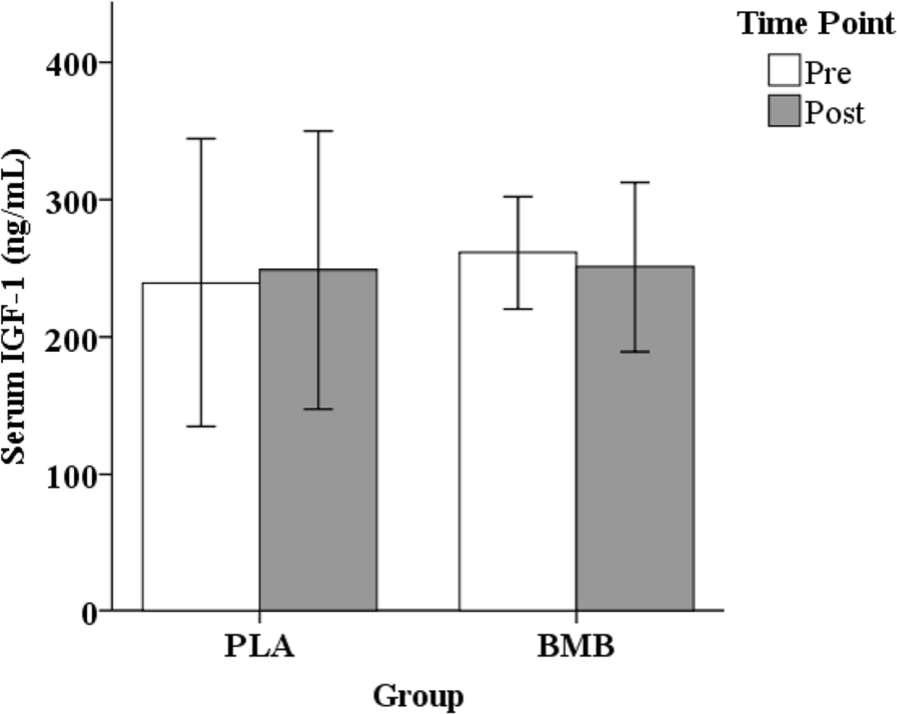
Mean ± standard deviation for resting serum insulin-like growth factor-1 (IGF-1) at the Pre and Post time points for each group. Note. Whisker bars represent the standard deviation; PLA = placebo; BMB = Bang Master Blaster. No statistically significant differences present

No significant interaction between group and time was observed for serum IGF-1 (*p* = 0.34; partial *n*^2^ = 0.44; Fig. 6). No significant main effect of time (*p* = 0.95; Cohen’s *d* = 0.01) or group (*p* = 0.77; Cohen’s *d* = 0.15) was observed.

### Skeletal muscle microRNA expression

**Fig. 7:**
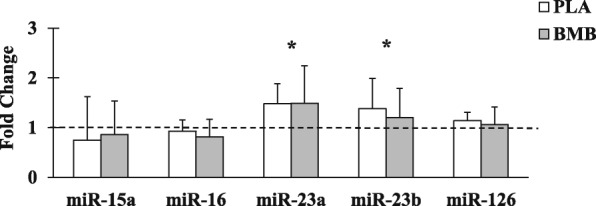
Average fold change in microRNA (miR) expression from baseline (represented by dashed line). Note. Whisker bars represent the standard deviation; PLA = placebo; BMB = Bang Master Blaster; * denotes statistically significant increase from Pre

No significant interaction effects between group and time were observed for miR-15 (*p* = 0.72; partial *n*^2^ = 0.01), miR-16 (*p* = 0.55; partial *n*^2^ = 0.03), miR-23a (*p* = 0.98; partial *n*^2^ < 0.01), miR-23b (*p* = 0.57; partial *n*^2^ = 0.03), or miR-126 (*p* = 0.71; partial *n*^2^ = 0.01) expression.. A significant main effect for time was observed for miR-23a (*p* = 0.01; Cohen’s *d* = 1.04) and miR-23b (*p* = 0.05; Cohen’s *d* = 0.70) expression with both significantly increased at Post compared with Pre. No significant main effect of time was observed for miR-15 (*p* = 0.24; Cohen’s *d* = 0.40), miR-16 (*p* = 0.21; Cohen’s *d* = 0.39), or miR-126 (*p* = 0.33; Cohen’s *d* = 0.36). The main of effect of group was not significant for miR-15 (*p* = 0.64; Cohen’s *d* = 0.17), miR-16 (*p* = 0.16; Cohen’s *d* = 0.51), miR-23a (*p* = 0.67; Cohen’s *d* = 0.16), miR-23b (*p* = 0.21; Cohen’s *d* = 0.47), or miR-126 (*p* = 0.39; Cohen’s *d* = 0.34; Fig. 7).

## Discussion

The main findings of the study were that 4 weeks of resistance training with ingestion of BMB was able to preferentially increase LBM and maximal strength compared with resistance training with ingestion of PLA without adversely affecting resting hemodynamics or blood safety markers. Our present data indicated a 5.9% increase in LBM in the BMB group and a 1.5% increase in LBM in the PLA group. These findings are slightly higher than similar previously completed 4-week MIPS studies utilizing NO-Shotgun® (NO) [16] and NO-Shotgun® in conjunction with NO Synthesize® (NOSS) [17], which found LBM increases of 4.8 and 3.7%, respectively. The greater increases in lean mass may be attributed to the higher dosages of key ingredients contained in BMB, such as betaine and creatine, than contained in NO-Shotgun®. Bench press 1-RM increased by 10.7% for the BMB group and by 4.8% for the PLA group in the current study. Similarly, bench press 1-RM increased 8.8 and 12.6% for the NO and NOSS studies, respectively [16, 17]. For lower-body strength, we observed a 16.1% increase in the BMB group and 10.7% increase in the PLA group for the box squat 1-RM. The previous studies observed lower-body strength increases of 18.4 and 21.3% for NO and NOSS, respectively [16, 17]; however, leg press 1-RM was utilized for those studies making the % increases not directly comparable to the current data.

Many potential mechanisms are responsible for the preferential increase in maximal strength and LBM in the BMB group. We previously demonstrated BMB to increase lower-body exercise performance after acute ingestion [10]. Enhanced acute exercise performance could have potentially led to a quicker accumulation of adaptations over the four-week training period. Acute caffeine supplementation appears to enhance power output and maximal strength which may lead to better performance during individual exercise sessions [18]. Caffeine may also reduce ratings of perceived exertion allowing for greater effort during each session which may be an important aspect of pre-exercise supplementation as previous studies indicate exercising to volitional fatigue is important for increases in hypertrophy [18, 19]. In addition to caffeine, beta-alanine is a precursor to carnosine which serves as a muscle buffer during intense exercise thus potentially increasing resistance exercise performance by reducing skeletal muscle fatigue and increasing work capacity [20]. It is unknown if beta-alanine contributes significantly to LBM or maximal strength, but its ability to increase training volume may potentially result in greater resistance training adaptations over time [20].

L-citrulline malate reportedly increases skeletal muscle protein synthesis by increasing L-arginine availability and through the mechanism of iNOS which activates mTOR signaling [21]. Yet, results of an 8-week study of L-citrulline supplementation combined with resistance training indicated no significant effect of supplementation on LBM [22]. Betaine anhydrous has been shown to enhance the anabolic endocrine response to acute resistance exercise along with increased myoblast differentiation [23, 24]. However, the overall results of studies utilizing betaine anhydrous to determine changes in strength and power are mixed [25]. A recent 8-week study in college-aged female participants did not result in a preferential increase in vertical jump or lower- or upper-body 1-RM measures, although a favorable increase in body composition was observed [26] which was in alignment with an earlier study in men performed by the same group [27].

Branched-chain amino acids (BCAAs) stimulate activation of the Akt/mTOR pathway resulting in increased muscle protein synthesis [28]. Yet, BCAA supplementation alone may not maximally increase muscle protein synthesis and support for their use as a stand-alone ergogenic aid is lacking [28, 29]. In contrast, BCAA in combination with a low dose of whey protein has been demonstrated to increase muscle protein synthesis rates to levels observed with 4x higher protein intake [30]. Specifically, leucine is likely the most important BCAA for supplementation [29]. Yet, leucine supplementation by itself has not been demonstrated to improve resistance training outcomes over extended periods of time [29, 31]. However, as already noted with the other ingredients, how BCAA supplementation is affected by co-ingestion of the multitude of other ingredients contained in BMB is unknown.

Creatine monohydrate is one of the most well-studied dietary supplements and has strong evidence to support its use to enhance resistance training adaptations [32]. Creatine monohydrate has been demonstrated to increase skeletal muscle stores of creatine and phosphocreatine, LBM, maximal strength, and work capacity, among other potential benefits [32]. Super Creatine™ (i.e. creatyl-L-leucine) is a novel ingredient consisting of creatine bound to L-leucine by a covalent bond and an initial toxicological evaluation in rodents demonstrated no genotoxic effects [33]. As this ingredient has never been independently evaluated as an ergogenic aid, it is impossible to determine to what extent it may have contributed to the beneficial effects of BMB.

Since BMB is a MIPS, it is not possible to determine which ingredients were directly responsible for the increased training adaptations or if there is a synergistic effect from the combination of the ingredients. Some of the ingredients contained in BMB have been previously implicated to increase adaptations to resistance training while data for other ingredients are mixed or lacking. Systematic addition and removal of ingredients over multiple studies are needed to determine the synergistic or stand-alone effect of each ingredient typically included in MIPS.

We found no evidence to support alterations in resting serum IGF-1 as a result of supplementation, resistance training, or a combination thereof. Our previous work demonstrated an acute increase in serum IGF-1 following an acute exercise bout with BMB supplementation, although the practical implications of that finding are unknown [10]. In contrast to our current finding, multiple previous studies demonstrate increased circulating IGF-1 with resistance training, with one similar four-week study increasing serum IGF by ~ 9% [16]. However, the role of serum IGF-1 in mediating skeletal muscle adaptations to resistance exercise is likely not as important as previously suggested [34], with multiple studies refuting its necessity for muscle hypertrophy [35, 36]. Morton et al. [36] reported that neither circulating nor intramuscular hormones, or the enzymes regulating their intramuscular production, influence skeletal muscle hypertrophy.

We observed training-induced increases in skeletal muscle basal expression of miR-23a and miR-23b. No changes were observed for miR-126, miR-16, and miR-15a. Although supplementation with BMB resulted in favorable strength and LBM changes, no differences were observed for the miR response. These specific miRs were chosen because of their ability to differentiate between powerlifters and sedentary controls in a previous study [5], suggesting their importance in resistance exercise adaptations, and because of their purported role in the regulation of proteins associated with anabolic and catabolic signaling within skeletal muscle. Skeletal muscle miR expression is altered in response to acute resistance exercise [37]. The current study design was not able to distinguish if BMB supplementation alters the acute response of the current miRs in response to exercise. Nonetheless, the finding of increased skeletal muscle miR-23a and miR-23b expression following 4 weeks of resistance training is important as it further suggests a role of these two miRs in the regulation of skeletal muscle adaptations as both have been shown to inhibit translation of atrogin-1 and MURF-1 [5, 38].

Regarding hemodynamic measures, resting blood pressure and heart rate were not altered in either condition. In our previous study, we also did not observe an increase in blood pressure or heart rate after acute consumption of BMB [10]. These results suggest BMB to be safe in terms of cardiovascular function; however, hemodynamic responses may be different in susceptible populations, such as obese persons or those with pre-existing health conditions. Further, hemodynamics during resistance exercise sets were not monitored, so it is possible that peak blood pressure and heart rate could be higher during these times. We did not observe any clinically meaningful alterations in whole blood or serum safety markers. The largest change observed was for serum creatinine levels in the BMB group; however, the amount of increase is consistent with previous studies involving creatine supplementation [39] and the mean value was within the normal clinical reference range. These data suggest no adverse effect of consuming BMB daily on the markers observed over a four-week period.

### Limitations

This study was limited by the short duration of resistance training; therefore, the current results cannot be extrapolated to longer periods of resistance training, i.e. 6 months to multiple years, after which adaptations may be more or less robust compared with placebo. The study is also limited by the inherent inaccuracies associated with dietary recalls [40]. The participants were asked to not change their dietary habits and to report all food intake for 3 days prior to each testing session. Although no differences were observed between groups or over time for macronutrient or kilocalorie intake, it is possible that dietary intakes were not reported accurately which could result in missed effects resulting from dietary intake. Furthermore, we could not mask the stimulant effects of caffeine in the BMB versus the PLA supplement.

Hemodynamic measurements were assessed at rest, which does not account for any potential alterations in heart rate or blood pressure experienced during exercise. Furthermore, similar to the hemodynamic measurements, blood and muscle samples were collected at rest. Consequently, only differences in basal levels of serum IGF-1 and miRs were studied. Changes in acute skeletal muscle miR expression in response to resistance exercise as a result of BMB supplementation may exist, as previously observed with serum IGF-1 [10], but they would be unable to be detected with the design of the current study. Lastly, the study is limited by a relatively small sample size. While the sample size of the current study was large enough to detect significant interaction effects regarding LBM and maximal strength, a larger sample size would give a better representation of the true change to be expected in the study population as individual responses to resistance training and supplementation present with wide variability [41].

## Conclusions

In conclusion, BMB supplementation combined with resistance exercise training for 4 weeks resulted in superior adaptations in maximal strength and LBM compared with resistance training with a placebo. No adverse resting hemodynamic or clinical blood safety markers were observed as a result of BMB supplementation. The superior outcomes associated with BMB supplementation could not be explained by resting serum IGF-1 or the skeletal muscle miRs measured, although resting miR-23a and miR-23b expression both increased as a result of resistance training. Future research should study the effects of BMB supplementation combined with resistance training over a longer training period to determine long-term effects on resistance training adaptations. Additionally, new studies utilizing a systematic method of adding and removing individual ingredients to determine the ergogenic effectiveness of each nutrient in combination with other commonly used nutrient in MIPS are warranted.

## Supplementary information

**Additional file 1: Table S1.** MicroRNA Primer Sequences.

## Data Availability

Additional data generated and analyzed during this study regarding serum brain-derived neurotrophic factor can be found using the following citation: Neil A. Schwarz, Sarah K. McKinley-Barnard, and Zachary J Blahnik. A randomized, double-blind, placebo-controlled trial of 4 weeks of resistance training combined with Bang® Master Blaster™ supplementation on lean body mass, maximal strength, mircoRNA expression, and serum hormones. Proceedings of the Fifteenth International Society of Sports Nutrition (ISSN) Conference and Expo Clearwater, FL USA. 6–8 June 2018.

## Abbreviations

1-RM: One-repetition maximum
ANOVA: Analysis of variance
BMB: Bang® Pre-Workout Master Blaster™
CBC: Complete blood count
CMP: Comprehensive metabolic panel
DXA: Dual-energy x-ray absorptiometry
IGF-1: Insulin-like growth factor-1
LBM: Lean body mass
MIPS: Multi-ingredient pre-workout supplements
miR: Microrna
MURF-1: Muscle RING-finger protein-1
PLA: Placebo
RT-PCR: Real-time polymerase chain reaction
TBM: Total body mass
