# Supplementary material for: Effect of Bang® Pre-Workout Master Blaster® combined with four weeks of resistance training on lean body mass, maximal strength, mircoRNA expression, and serum IGF-1 in men: a randomized, double-blind, placebo-controlled trial
Source: J Int Soc Sports Nutr. 2019 Nov 19;16:54. doi: 10.1186/s12970-019-0310-y (PMC6862793; doi:10.1186/s12970-019-0310-y)
Supplement: Supplementary file 1 — Additional file 1: Table S1. MicroRNA Primer Sequences. [file 12970_2019_310_MOESM1_ESM.docx]

| **Additional file 1: Table S1.** MicroRNA Primer Sequences. | | | |
| --- | --- | --- | --- |
| **microRNA ID** | **Accession** | **Human microRNA Sequence** | **Primer Sequence** |
| hsa-miR-15a-5p | MIMAT0000068 | UAGCAGCACAUAAUGGUUUGUG | TAGCAGCACATAATGGTTTGTGA |
| hsa-miR-16-5p | MIMAT0000069 | UAGCAGCACGUAAAUAUUGGCG | CGCCATAGCAGCACGTAAAT |
| hsa-miR-23a-5p | MIMAT0004496 | GGGGUUCCUGGGGAUGGGAUUU | GTTCCTGGGGATGGGATTTA |
| hsa-miR-23b-5p | MIMAT0004587 | UGGGUUCCUGGCAUGCUGAUUU | GGTTCCTGGCATGCTGAT |
| hsa-miR-126-3p | MIMAT0000445 | UCGUACCGUGAGUAAUAAUGCG | CGTACCGTGAGTAATAATGCGAAA |
| hsa-miR-186-5p | MIMAT0000456 | CAAAGAAUUCUCCUUUUGGGCU | CAAAGAATTCTCCTTTTGGGCTA |
| hsa-miR-320a | MIMAT0000510 | AAAAGCUGGGUUGAGAGGGCGA | CTGGGTTGAGAGGGCGAAA |
| hsa-miR-361-5p | MIMAT0000703 | UUAUCAGAAUCUCCAGGGGUAC | TCAGAATCTCCAGGGGTACAA |
